# Five Years Experience of *Clostridium difficile* Infection in Children at a UK Tertiary Hospital: Proposed Criteria for Diagnosis and Management

**DOI:** 10.1371/journal.pone.0051728

**Published:** 2012-12-26

**Authors:** Sumita Pai, Sani Hussaini Aliyu, David Andrew Enoch, Johannis Andreas Karas

**Affiliations:** 1 Clinical Microbiology & Public Health Laboratory, Health Protection Agency, Addenbrookes Hospital, Cambridge, United Kingdom; 2 Peterborough & Stamford Hospitals NHS Foundation Trust, Peterborough City Hospital, Bretton Gate, Peterborough, United Kingdom; Beijing Institute of Microbiology and Epidemiology, China

## Abstract

**Background:**

*Clostridium difficile* infection (CDI) is associated with significant morbidity and mortality in adults. There is increasing evidence of the pathogenic role of *C. difficile* in the paediatric population. We sought to ascertain the clinical presentation and severity of CDI in children at our institution and develop criteria to aid management.

**Methods:**

Clinical data was retrospectively collected from all children (0–16 yrs) with a positive *C. difficile* toxin result over a 5-year period. National adult guidelines were used to assess the severity and management of CDI.

**Results:**

Seventy-five patients were included with a mean age of 2.97 years. Forty-nine were hospital onset, 22 community onset and 4 healthcare-associated. The most common co-morbidity among the hospital onset infections was malignancy. Gastrointestinal conditions were most common among community onset infections. Fifty-five cases (73.3%) had received antibiotics in the preceding month, 7 (9.3%) had cow’s milk intolerance and 9 (12%) had co-infection with another gut pathogen. According to national adult guidelines 57 cases (76%) were categorised as severe. Thirty cases received oral metronidazole, two patients required intensive care and one patient had a sub-total colectomy for pseudomembranous colitis. No mortality was observed.

**Discussion:**

We confirm the association of paediatric CDI with co-morbidities such as haematological and solid organ malignancies, recent antibiotic use and hospitalisation. We observed an association between cows milk protein intolerance and *C. difficile.* The use of adult criteria overestimated severity of disease in this cohort, as most cases experienced a mild course of illness with low morbidity and no mortality. This indicates that adult scoring criteria are not useful in guiding management and we propose specific criteria for children.

## Introduction

*Clostridium difficile* (*C. difficile*) is the commonest cause of hospital acquired diarrhoea in adults and is associated with significant mortality and morbidity. [1] The role of *C. difficile* in children is less certain. The convention is to regard *C. difficile* in stools in children less than two years of age to represent colonisation (i.e. not a cause of diarrhoea), although severe disease does occur. The mandatory surveillance in England and Wales does not include children less than two years of age and many laboratories do not test samples from children. Numerous surveys and studies looking at the significance of isolating *C. difficile* in children show increasing evidence for a potential pathogenic role in the paediatric population. [2]–[4] *C. difficile* is also becoming increasingly recognised as a significant co-morbid factor in prominent paediatric inpatient populations such as oncology [5]–[8] and with patients with gastrointestinal disorders. [9], [10] Community onset *C. difficile* infection (CDI) without previous direct or indirect contact with a hospital environment remains rare compared with hospital onset CDI. Nevertheless, it has been reported in populations that were previously thought to be at low risk, such as young individuals and pregnant women [11].

A recently published literature review looking at colonisation and disease in children with *C. difficile* showed that *C. difficile* is frequently isolated from children of all ages and colonisation appears to occur soon after birth and rises to high levels at one year. High carriage rates are associated with hospitalisation. CDI was strongly linked to antibiotic associated diarrhoea in children but symptoms were more likely to be severe in the presence of co-morbidities such as malignancy and immunosuppression [12].

The current UK Department of Health (DH) & Health Protection Agency (HPA) guidelines for clinical diagnosis has an adult focus and prompts testing for *C. difficile* when there is a history of abdominal pain, profuse, foul-smelling, soft stools, and fever. [13] These features are not specific but should be taken into context with recent antibiotic use and/or stay in hospital. [11] It has been suggested that leucocytosis is particularly prominent in CDI, but again this alone is inadequate for diagnosis. Radiological imaging of the abdomen is non-specific. [13] Management is based on severity of infection with oral metronidazole as the recommended first line treatment in mild to moderate cases and vancomycin used or added in severe cases. Colectomy is considered in life-threatening disease [13].

The aim of this study was to ascertain the clinical presentation and severity of disease in children with a positive *C. difficile* toxin result over a 5 year period at our institution and to develop criteria to aid diagnosis and management.

## Methods

Addenbrooke’s Hospital is a tertiary centre covering both rural and urban populations. Paediatric care includes neonatal and paediatric intensive care units (ICU), general medical and surgical beds and a specific haematology/oncology ward. Addenbrooke’s has 81 paediatric beds and in 2010 it treated 3,442 paediatric day cases and 3,844 paediatric inpatients.

We performed a retrospective descriptive study of all patients with a positive *C. difficile* toxin result over a 5-year period, from 1st January 2005 to 31^st^ December 2009. The study was identified as a service evaluation and approval to carry out the study was obtained from the trust’s audit committee. Research ethics was not required.

We followed the UK HPA Steering Group on Healthcare Associated Infection (2008) guidance for testing *C. difficile* in laboratories. [13] These guidelines recommend diarrhoea to be defined as stool loose enough to take the shape of the container, or as Bristol Stool Chart types 5–7. [14] All samples from patients ≥2 years of age were routinely tested for *C. difficile* toxin. Patients under two were tested only on the specific request of paediatricians and following discussion with the laboratory. Samples classified as Bristol Stool Chart type 1–4 were tested only after discussion between the paediatricians and the laboratory on an individual basis. *C. difficile* testing was performed by EIA (VIDAS, BioMerieux, Basingstoke, UK) followed by confirmation with cell cytotoxin assay (Vero cell line). Samples were not retested within 28 days of a positive result. A positive *C. difficile* toxin test ≥28 days after the initial positive assay was defined as a recurrence in line with DH criteria. [13] Samples were also tested at the same time for bacteriology (*Campylobacter, Salmonella, Shigella, E. coli* 0157) using HPA methodology, parasitology (*Cryptosporidium*) by immunofluorescent staining and viruses.

Hospital onset infection was defined as a positive test after two days of hospital admission. Community onset was defined as a positive test within two days of admission and healthcare-associated if patients came from nursing homes or palliative care facilities.

Information that was collected for each patient included demographics, past medical history, risk factors, laboratory diagnosis, severity score, clinical management and outcome at 3 months. Clinical data were obtained from medical, nursing, pharmacy and microbiology records and laboratory data was obtained from the laboratory information management system. *P*-values were calculated for the following – age distribution and co-morbidities (gastro-intestinal, haematology, immunosuppression and solid organ malignancy) using chi-squared test the via online GraphPad software.

Disease severity was classified using the Department of Health criteria of mild, moderate, severe and life-threatening infection. [13] Mild CDI was defined as typically associated with <3 stools of types 5–7 on the Bristol Stool Chart per day with a normal WCC, moderate CDI is associated with a raised WCC that is <15 x109/L and typically associated with 3–5 stools per day, severe CDI is associated with a WCC >15 x109/L, or an acute rising serum creatinine (i.e. >50% increase above baseline), a temperature of >38.5°C or evidence of severe colitis (abdominal or radiological signs), whilst features of life-threatening CDI included hypotension, partial or complete ileus or toxic megacolon, or CT evidence of severe disease. These guidelines also recommend treating according to severity, daily monitoring using the Bristol Stool Chart and multidisciplinary clinical review of cases.

## Results

Eighty nine patients had a positive *C. difficile* toxin result over the 5 years. Data could not be found on 14 patients and were excluded, leaving 75 patients for analysis. There were 27 cases in 2005, 15 in 2006, 20 in 2007, 9 in 2008 and 4 in 2009. The mean age was 2.97 years and the median age was 2 years (range 2 days to 14 years). Thirty-six (48%) patients were below the age of 2 years. Thirty-seven (49.3%) patients were female. Of the 75 cases, 49 (65.3%) were hospital onset, 22 (29.3%) community onset and four (5.3%) were healthcare-associated. The mean in-patient days prior to *C. difficile* being isolated from stools was 27.2 days with a median of 10 days (range 0 to 213 days) for the hospital onset cases. Thirty-one (58.5%) of the hospital onset cases had prior in-patient days of >1 month in the preceding year before isolation of *C. difficile* from stools. Detailed results comparing hospital and community onset cases are presented in table 1.

**Table 1:** Results comparing hospital onset with community onset cases.

		Hospital associated (%)(N = 53)	Community associated (%) (N = 22)	Total (%)(N = 75)	p- values
**Age (yrs)**	<2 years	16 (30.2)	20 (90)	36 (48)	0.0001
	2–5 years	26 (49)	1 (4.5)	27 (36)	
	>6 years	11 (20.7)	1 (4.5)	12 (16)	
Gender	Male	25 (47.2%)	12 (54.5%)	37 (49.4%)	
Year	2005	14	13	27	
	2006	11	4	15	
	2007	15	5	20	
	2008	9	0	9	
	2009	4	0	4	
**Co-morbidities** *	Cardiovascular	3 (5.7)	0	3 (4)	
	Neuromuscular	3 (5.7)	1 (4.5)	4 (5.3)	
	Gastrointestinal	3 (5.6)	5 (22.7)	8 (10.7)	<0.0001
	Haematology + immunosuppression	11 (20.7)	1 (4.5)	12 (16)	0.0001
	Malignancy (solid organ)	20 (37.7)	0	20 (26.7)	0.0001
	Renal+Metabolic	3 (5.6)	0	3 (4)	
	Cows milk allergy	1 (1.8)	6 (11.3)	7 (9.3)	<0.0001
	Respiratory	1 (1.8)	1 (4.5)	2 (2.7)	
	Others	7 (13)	3 (13.6)	10 (13.3)	
**Antibiotic history**	Penicillins (Amoxicillin & Co-amoxiclav)	5 (9.4)	5 (22)	10 (13.4)	
	Cephalosporins	27 (50.9)	3 (13.6)	30 (40)	
	Quinolones (Ciprofloxacin)	19 (35.8)	0	19 (25)	
	Others	5 (9.4)	2 (9)	7 (9.3)	
	No antibiotics	7 (13.2)	13 (59)	20 (26.7)	
**Co-infections**	Bacterial	0	0	0	
	Viral	6 (11.3)	3 (13.6)	9 (12)	
**Other risk factors**	Intensive Care stay	12 (22.6)	0	12 (16)	
	Surgery**	14 (26.4)	4 (18)	18 (24)	
**Severity score** ***	Mild	7 (13.2)	2 (9)	9 (12)	
	Moderate	6 (11.3)	2 (9)	8 (10.7)	
	Severe	39 (73.6)	18 (81.8)	57 (76)	
	Life threatening	1 (1.9)	0	1 (1.4)	
**Treatment**	Not required	27 (51)	13 (59)	40 (53.4)	
	Metronidazole	22 (41.5)	8 (36.4)	30 (40)	
	Escalation of treatment to Vancomycin	3 (5.7)	0	3 (4)	
	Probiotics	1 (1.9)	1 (4.5)	2 (2.7)	
**Outcome**	Alive at 3 months	45 (81)	19 (81.8)	64 (81.4)	
	ICU requirement	2 (2.8)	0	2 (2.7)	
	Colectomy	1 (1.8)	0	1 (1.3)	
	Crude mortality	2 (3.8)	2 (9)	4 (5.4)	
	Missing notes	2	1	3	
	Transferred	3	1	4 (5.4)	

* Note some patients had more than one risk factor.

** Of the 18 surgeries 9 were gastrointestinal surgeries (Resection of abdominal mass, appendicectomy).

*** Severity scoring criteria as recommended by Department of Health and Health Protection Agency.

Sixty-eight patients (90.7%) had significant co-morbidities (table 1). Among the haematology patients, 7 had acute lymphoblastic leukaemia, three had acute myeloid leukaemia and two had non-Hodgkins lymphoma. Seven patients (9.3%) were diagnosed with possible cows milk protein allergy.

Four patients had previous CDI prior to 2005 and two patients had recurrences. Both recurrences occurred in haematology patients with prolonged stay in hospital.

Twenty patients (26%) had no exposure to antibiotics in the preceding month. Six patients had ≥2 antibiotics in the preceding month. Antibiotics most frequently implicated included 3^rd^ generation cephalosporins (30) and fluoroquinolones (19), but also included flucloxacillin, piperacillin-tazobactam, meropenem, vancomycin and gentamicin (table 1). Five (7%) patients were on PPIs prior to the onset of symptoms and 19 (25%) had recently received H_2_ receptor antagonists. Other risk factors are shown in table 2.

**Table 2:** Blood parameters and symptoms of infection for symptomatic patients (n = 68).

Parameter		Number of patients (%)
Blood parameters at CDT date	WCC (>15×10^9^)	28 (41.2)
	Rising Creatinine	2 (2.9)
	Falling Albumin (<25 g/L)	10 (14.7)
	CRP (>25 mg/L)	40 (58.8)
	Hb (<10 g/L)	28 (41.2)
Fever ≥38.5°C		34 (50)
Abdominal pain, tenderness, distension		17 (25)
Diarrhoea >5 times a day		50 (73.5)
Pseudomembranous colitis		2 probable* 1 definite
Surgery required		1

* In two cases the endoscopy results was unable to discriminate between typhlitis and pseudomembranous colitis.

Key: WCC – white cell count; CRP – C-reactive protein; Hb – haemoglobin; CDT – *Clostridium difficile* toxin.

Virology tests were performed for 51 cases; three were co-infected with adenovirus, three with norovirus, two with rotavirus and one with enterovirus. No additional bacterial infection was found in any of the cases.

Nine patients were considered to have mild infection, 8 moderate, 57 severe and one patient had life-threatening disease according to adult parameters devised by the DH/HPA.13 The findings of blood parameters and symptoms of infections are summarised in table 3.

**Table 3:** Proposed revised criteria for severity of *Clostridium difficile* infection in children.

Criteria	Point
Diarrhoea >5 times a day	1
Abdominal pain and discomfort	1
Rising white cell count	1
Raised C-reactive protein	1
Pyrexia >38°C	1
Evidence of pseudomembranous colitis	2
Intensive care unit requirement	2

Score

1–2 =  mild disease.

3–4 =  moderate disease.

≥5 =  severe disease.

Seven patients (9.3%) were tested with a sample consistent with Bristol stool chart of 1–4.Treatment was deemed unnecessary in a further 33 patients (44%) by their respective clinical teams. Thirty (40%) cases were treated with metronidazole alone and two received probiotic treatment only. No patient received cholestyramine. Metronidazole usage led to symptom resolution in a mean of two days. Escalation of treatment was required in three cases with oral vancomycin being added to metronidazole.

In nine patients preceding broad spectrum antibiotics were continued despite ongoing diarrhoea. Laxatives were not stopped in 6 patients. A stool chart was kept only in 26 (34.7%) cases throughout the symptomatic period.

Outcome data are shown in table 1. One case developed pseudomembranous colitis (diagnosed endoscopically) and required a sub-total colectomy. Thirty-five (46.7%) cases required admission or prolongation of their hospital stay due to CDI. Four patients died (5.3%). One was thought to be due to a combination of significant bacteraemia along with ongoing diarrhoea and the other three died due to their underlying disease.

Using our proposed criteria (table 3), of the 68 symptomatic cases in our study, 54 fulfilled the diagnosis of likely CDI. Of these 54; 26 (48%) would have been categorised as having mild disease (versus 9 using adult criteria), 23 (42.6%) moderate disease (versus 8), 5 (9%) severe disease (versus 57) and none with life-threatening disease (versus one). The severity numbers are lower and befitting the observations from our study.

## Discussion

We describe the presentation and severity of *Clostridium difficile* in children. We found that a significant number of patients did not require treatment and the overall morbidity and mortality rates were low, despite most being categorised as “severe” by the UK DH adult standards. The majority (49.3%) of cases were hospital onset, most had received antibiotics and most (59.6%) were undergoing or had already received chemotherapy for haematological or solid organ malignancies. Of the community onset cases, most had a past history of gastrointestinal conditions including cow’s milk protein allergy, gastro-oesophageal reflux disease or recent gastrointestinal surgery. We found a reduction in the total number of cases from 2005 to 2009. This was similar to observed trends in adult patients with CDI in our centre and likely reflects enhanced infection control measures including early isolation of patients, enhanced environmental cleaning and antibiotic stewardship. There was no change in laboratory test methods during this period.

Over the 5-year period to 2006, a large US study by Kim and colleagues reported a steady increase in the annual incidence of CDI amongst paediatric inpatients, from 2.6 to 4 cases per 1000 admissions. The median age of children with CDI was 4 years. They also noted an increase in CDI cases in the 5–17 year age groups over this time period. [15] In contrast, our study demonstrated a younger median age of 2 years (mean was 2.97 years) and there was no increase in cases in the 5–17 year age groups.

Seven of our cases were asymptomatic, but were tested on special request and therefore did not fit the DH criteria [13] for diagnosis of CDI; colonisation was thought to be more likely among these patients.

CDI appears to be strongly linked to the presence of co-morbidities such as haematological malignancies, immunosuppression and bowel disorders. [7], [10], [16]–[19] In our study, among the 68 patients with significant co-morbidities 12 (17.6%) had haematological malignancies and 20 (29%) had solid organ tumours. Both groups of patients were receiving or had previously received chemotherapy. These groups also had the highest rate (59.5%, p value of 0.0001) of hospital onset *C. difficile*. Other studies have similarly found increased rates of CDI in these patient groups. [7], [18], [20] Haematology/oncology patients are recognised as a frequent source of *C. difficile* in hospitals amongst the paediatric population. [5], [6], [16], [17], [19] This could be due to a combination of factors such as frequent hospital encounters, prolonged antibiotic usage (these patients had the most number of antibiotic prescriptions) and immunosuppression (62.5% were neutropenic). Neutrophils have been implicated as playing a pivotal role in the pathophysiology of CDI [19].

Gastrointestinal co-morbidities were more common in community onset CDI (45%; p value of <0.0001). Seven out of the ten cases were being investigated for cow’s milk protein allergy (CMA) along with multiple food allergies. Only one of these 7 cases had prior exposure to antibiotics (amoxicillin) and had loose stools prior to starting antibiotics. An association has been described between gut colonisation with *Clostridium difficile* and allergy in children [21].

Antibiotic usage is a widely recognised major risk factor for development of CDI. [16], [22], [23], [24], [25] In our study, 55 patients (74%, p value 0.0001) had received antibiotics in the previous month, of which 50 (91%) were symptomatic with profuse diarrhoea (more than 5 times a day). Cephalosporins were the most commonly used antibiotics but this may reflect greater use in children overall. Ciprofloxacin was the second most frequently used antibiotic, mainly in the haematology and oncology patients.

Exposure to stomach acid-reducing agents, such as H_2_ receptor antagonists and PPIs, remains a controversial risk factor, and has been associated with CDI in some studies [22] but not in others. [11], [26] Only five (7%) of our patients were on PPIs prior to the onset of symptoms and 19 (25%) had recently received H_2_ receptor antagonists.

To date, the significance of co-infection with another gut pathogen is uncertain. In our study, nine patients had evidence of co-infection; three of these had community onset diarrhoea (two rotavirus and one norovirus) and the other six were hospital onset (three adenovirus, one enterovirus and two norovirus). Two of the three cases with norovirus co-infection were symptomatic and settled eventually after four days without treatment. Lukkarinen and colleagues hypothesized in two cases that norovirus may affect epithelial homeostasis of intestine and exacerbate the effects of toxins produced by *C. difficile* ribotype 027 [27].

All patients were reviewed on a daily basis by their respective clinical teams. In the majority of cases the clinical teams did not classify patients according to severity of disease. In ten cases (13.3%) the clinical teams judged the infection to be severe; 7 improved with metronidazole alone whilst the remaining three received additional vancomycin due to non-resolution of symptoms. Of the three cases that received dual antibiotic therapy, two required ICU admission for monitoring of their renal function and another had disease severe enough to require a subtotal colectomy. In contrast, using national DH/HPA adult criteria an additional 47 cases would have fallen into the severe category of disease. Examining these 47 cases, 18 spontaneously recovered without active treatment and two received probiotic treatment alone. The remaining 27 cases received metronidazole with three requiring additional vancomycin and ICU input in two cases. The commonest symptoms and signs among our cohort were diarrhoea, a rising CRP, white cell count >15×109/L and pyrexia >38.5°C. These signs and symptoms are included in the DH description of severe CDI. The DH/HPA adult guidance recommends treatment of severe disease with oral vancomycin initially and adding IV metronidazole if no improvement is observed, which differs to the treatment received by our patients.

The majority of the patients in our study had a favourable outcome. The primary outcome was all-cause 3-month mortality rate. There was no *C. difficile* related death in our cohort and morbidity was low. From published studies the mortality and morbidity data due to CDI in children is low [15], [16], [25].

The above findings highlight three important observations of CDI in children. Firstly, children appear to have a mild course of disease despite having an acute response physiologically to infection. This would explain the discrepancy between the observed course of disease and that predicted by the DH/HPA adult severity scoring system (which uses acute response as a predictor for disease prognosis). Secondly, most of our cases had an overall good outcome with either no treatment or oral metronidazole. Lastly, the intuitive clinical triaging used by the teams in our study appears to be more relevant to the paediatric population in grading patient severity.

Based on the results of our study, we propose the following guidance for CDI in children in an attempt to help differentiate between *C. difficile* colonisation and infection and secondly, a scoring system to accurately grade severity and guide appropriate treatment.

### Proposed Diagnostic Criteria

1. Fulfils likely diagnosis of CDI if – Diarrhoea (Bristol stool chart 5–7) **and** one or more of the following:

- Significant co-morbidities – Haematology/oncology, Gastrointestinal disease
- Stay in hospital for >2 days
- Antibiotic use in the last 1 month (especially ciprofloxacin and cephalosporins)

Our revised criteria for severity of infection are described in table 3.

### Proposed Medical Management

#### Mild disease

No need to treat if symptoms settle within 24 hours but consider oral metronidazole for 10–14 days if symptoms persist beyond 24 hours.

#### Moderate disease

Oral metronidazole for 10–14 days and consider escalation by changing to oral vancomycin if non-resolution of symptoms or decline in severity score.

#### Severe disease

Oral vancomycin and IV metronidazole. Colectomy should be considered if there if evidence of caecal dilatation.

Our intention is to carry out a prospective study using our proposed criteria to see whether it provides a consistent and beneficial tool towards the management of CDI in children.

In conclusion, from our study we found that applying the adult DH/HPA *C. difficile* severity scoring criteria overestimates the number of severe cases and is not a reliable guide for management in children. Most children in our study had a self-limiting course of illness regardless of treatment. We confirmed the association of CDI with co-morbidities such as haematology/oncology malignancies, recent antibiotic use and hospitalisation. We also observed a possible link between gastrointestinal disorders like cows milk protein intolerance and *C. difficile* but this area requires further investigation. We have proposed a diagnostic and severity scoring system to guide appropriate management.
